# Micro-RNA-195 and -451 Regulate the LKB1/AMPK Signaling Axis by Targeting MO25

**DOI:** 10.1371/journal.pone.0041574

**Published:** 2012-07-23

**Authors:** Hao Chen, Gustavo M. Untiveros, Laurel A. K. McKee, Jessica Perez, Jing Li, Parker B. Antin, John P. Konhilas

**Affiliations:** 1 Department of Physiology, University of Arizona, Tucson, Arizona, United States of America; 2 Department of Molecular and Cellular Biology, University of Arizona, Tucson, Arizona, United States of America; 3 Department of Cellular and Molecular Medicine, Molecular Cardiovascular Research Program and the Sarver Heart Center, University of Arizona, Tucson, Arizona, United States of America; 4 Department of Pediatrics, Steele Children’s Research Center, Tucson, Arizona, United States of America; Virginia Commonwealth University Medical Center, United States of America

## Abstract

**Background:**

Recently, MicroRNAs (miR) and AMP-kinase (AMPK) have emerged as prominent players in the development of cardiac hypertrophy and heart failure. We hypothesized that components of the adenosine monophosphate-activated kinase (AMPK) pathway are targeted by miRs and alter AMPK signaling during pathological cardiac stress.

**Methodology/Principal Findings:**

Using a mouse model of hypertrophic cardiomyopathy (HCM), we demonstrated early elevation of miR-195 and miR-451 in HCM hearts, which targets MO25, a central component of the MO25/STRAD/LKB1 complex that acts as an upstream kinase for AMPK. We show functional targeting of MO25 by miR-195 and -451. Further *in vitro* interrogation of MO25 as a functional target validated this hypothesis where over-expression of miR-195 in C2C12 cells knocked down MO25 expression levels and downstream AMPK signaling (phosphorylation of Acetyl CoA carboxylase [ACC] and AMPK activity assay), similar to MO25 knockdown in C2C12 cells by siRNA. Parallel changes were measured in 60 day R403Q HCM male hearts that were rescued by short-term administration of AICAR, an AMPK agonist.

**Conclusions/Significance:**

Elevated miR-195 targets the LKB1/AMPK signaling axis in HCM progression and implicates a functional role in HCM disease progression. MiR-195 may serve as potential therapeutics or therapeutic targets for heart disease.

## Introduction

MicroRNAs (miR) are small, noncoding RNAs 18–25 nucleotides (nt) in length that negatively regulate gene expression in a sequence-specific manner. Apart from a post-transcriptional role in gene expression, miRs regulate diverse biological and pathological processes, including cell proliferation, differentiation, apoptosis, carcinogenesis, embryogenesis, and immunity [1]–[3]. Recently, miRs have emerged as prominent players in the development of cardiac hypertrophy and heart failure [4]–[6] and may serve as potential therapeutics or therapeutic targets for heart disease [7]–[9]. For example, genetic deletion of miR-208 in the heart prevents the pathological sequelae associated pressure overload [10]. Similarly, systemic inhibition of miR208a by an antisense oligonucleotide improves cardiac function in a rat model of heart failure [11].

Recent studies show that AMP-kinase (AMPK) is a critical regulator of cellular metabolism and cardiac hypertrophy [12]–[14]. AMPK is a heterotrimeric enzyme complex consisting of a catalytic α subunit and regulatory β and γ subunits. Direct phosphorylation at Thr-172 (α subunit) by upstream AMPK kinases (AMPKKs) is required for activation and is a key mechanism by which cardiac AMPK is activated during times of metabolic stress. So far, only two AMPKKs have been identified in the heart: the tumor suppressor kinase LKB1 [15], [16] and a calmodulin-dependent protein kinase kinase (CamKK) [17]. The LKB1 complex consists of LKB1 and two accessory subunits, STRAD (Ste20-related adaptor) and MO25 (mouse protein 25; CAB39) both of which are required for full LKB1 activity [15], [16], [18], [19]. Additionally, in response to increases in intracellular calcium concentration during cellular stress, CaMKK can also phosphorylate Thr172 and activate AMPK. Activation of AMPK turns off energy consuming processes, such as protein synthesis, while switching on ATP-generating mechanisms, such as fatty acid oxidation (FAO) and glycolysis [20]. The combined effect of increased glycolysis, fatty acid oxidation and its ability to up-regulate mitochondrial biogenesis [21] is a net increase of oxidative ATP production.

Here we hypothesize that miRs regulate the AMPK signaling axis. To identify putative AMPK-target specific miRs, we performed a real-time PCR screen using the R403Q transgenic mouse model of HCM to identify disease-associated miRs [22], [23]. R403Q HCM mice express a mutant myosin heavy chain (R403Q) corresponding to a human mutation causing HCM and possess multiple phenotypic similarities with their human counterparts [22], [24]. More importantly, this R403Q model also demonstrates the energetic abnormalities that occur in cardiac disease states [25], [26]. In this study, we show that miR-195 and -451 are up-regulated in R403Q HCM male hearts, which targets MO25, a central component in the LKB1/AMPK signaling pathway [27] We further demonstrate that MO25, a central component of the LKB1/AMPK signaling axis, is a functionally relevant target of miR-195 and -451. These findings provide novel insight into the regulation of metabolic pathways during the pathological progression of cardiac disease and identify miR-195 and-451 as a potential therapeutic target for treatment of heart disease.

## Results

### Cardiac-specific Expression of miR-195 and -451 by RT-PCR and Northern Blot

**Figure 1:**
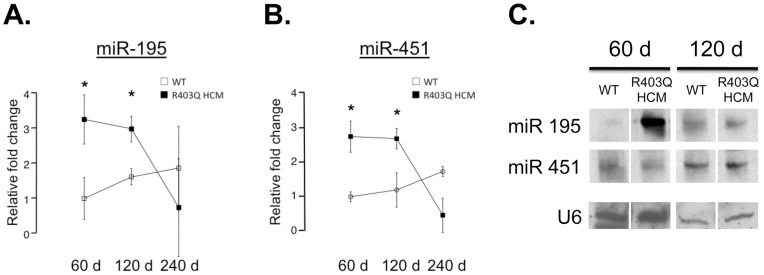
miR-195 and miR-451 expression in WT and R403Q HCM hearts. **A:** Expression levels determined by RT-PCR of miR-195; B: miR-451 (right panel) in 60, 120, and 240 day male WT and R403Q HCM hearts corrected by U6 expression (n = 3 for each group, *p<0.05), **C:** Northern blot analysis of miR-195 and miR-451 expression in WT and R403Q HCM hearts. Northern blot analysis using LNA-modified, 5′ end biotin-conjugated prob1es for U6 RNA, miR-195 and miR-451 using RNA extracted from 60-day- and 120-day-old WT and R403Q HCM hearts. Each lane represents one sample of extracted RNA pooled together from 4 total hearts. Northern blot images in C are taken from the same blot but were cropped for clarity.

To investigate the role of microRNAs (miRs) in the development of pathological cardiac hypertrophy, we performed a real-time PCR screen using the R403Q transgenic mouse model of hypertrophic cardiomyopathy (HCM) [22], [23]. This screen included 22 candidate miR targets implicated in pathological cardiac disease and/or metabolic dysregulation [6], [10], [28]. The hearts from male R403Q HCM mice were compared with sex- and age-matched wild-type (WT) littermate controls at three different timepoints (60, 120 and 240 days) corresponding to distinct stages in HCM disease progression [29], [30]. All expression levels were normalized to U6 snRNA and compared to WT controls at 60 days of age (Supplemental Figure S1). As shown in Figure S1, our candidate screen indicated that miR-195 and miR-451 were among the early-elevated miRs. Real time PCR results demonstrated a 3.2 fold elevation of miR-195 and a 2.9 fold elevation of miR-451 in R403Q HCM versus wild-type hearts at 60 days (Figure 1A). Elevated expression levels of both miR-195 and miR-451 persisted at 120 days but returned to control levels at 240 days. The relative expression of miR-195 was about eight-fold higher than miR-451 (data not shown).

### The Candidate Screen also Illustrated that the miR Expression Profile was Dependent on Stages of Disease Progression

During early stages of the HCM disease progression (60 or 120 days), elevated miRs include miR-27, -29, -195, -199, -208a, -208b and -451. At this same time point, suppression of miR-125 and -150 was observed with no significant change in miR-499 (Supplemental Figure S1). Late stages of HCM disease (240 days) [22], [23] corresponded largely with an attenuation of candidate miR expression (Supplemental Figure S1). Interestingly, miR 122, a predominant liver miR involved in lipid metabolism [28] was present in cardiac tissue and elevated at this late time point.

The elevation of miR-195 has been observed in hypertrophic mouse hearts induced by thoracic aortic banding (TAB), transgenic mice expressing activated calcineurin A (CnA) in the heart, and in human failing hearts [6]. Moreover, overexpression of miR-195 results in severe cardiac hypertrophy and dysfunction [6]. Recently, miR-451 was reported to be downregulated during ischemia-reperfusion but upregulated in human infarcted hearts [31]. To validate the RT-PCR results for miR-195 and -451, Northern blot analysis of pooled RNA extracts from WT and R403Q HCM hearts (4 hearts from each group) confirmed the presence of miR-195 and miR-451 in cardiac tissue from both WT and R403Q HCM mice (Figure 1C). In addition, miR-195 levels observed by Northern blot paralleled the RT-PCR expression pattern in R403Q HCM male hearts (Figure 1C). The elevated expression of miR-451 in R403Q HCM hearts compared to controls observed by RT-PCR was validated by Northern blot only in 120-day hearts; there were no measurable differences measured by Northern blot at 60 days (Figure 1C). This may reflect the 8-fold lower expression level of miR-451 compared to miR-195, similar to that observed by RT-PCR.

### Cardiac-specific Expression of miR-195 and -451 by *in situ* Hybridization

**Figure 2:**
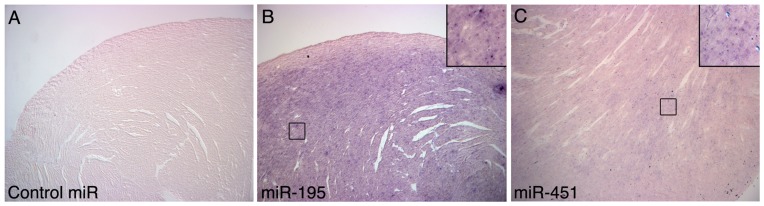
*In situ* hybridization of miR-195 and miR-451 expression in R403Q HCM hearts. In situ hybridization of miR-195 and miR-451 in the left ventricle of male R403Q HCM hearts. 5′ digoxin-conjugated LNA-modified DNA probes complementary to mature miR-195 or -451 were used to detect native miR-195 and -451. **Panel A** (100X magnification): Scrambled sequence, missense 5′ digoxin-conjugated LNA-modified DNA probe; **Panel B** (100X magnification): miR-195, 5′ digoxin-conjugated LNA-modified miR-195 sequence probe; **Panel C** (100X magnification): miR-451, 5′ digoxin-conjugated LNA-modified miR-451 sequence probe. **Insets:** 200 X magnification. No significant signal was detected in hearts probed with the scrambled sequence. Sections treated with probes specific for miR-195 or miR-451 demonstrated a detectable signal.

Because miR-451 shows high expression during erythrocyte maturation [32], we could not rule out that the detection of miR-451 by RT-PCR was due to red blood cell contamination in our whole heart preparations. Therefore, to validate miR-195 and -451 localization and visualize distribution in R403Q HCM hearts, we performed in situ hybridizations using LNA-modified probes for mature miR-195 and -451 in fixed sections of R403Q HCM hearts. Scrambled sequence miR probes were used as controls. As shown in Figure 2, R403Q HCM hearts showed positive staining for both miR-195 and -451. The scrambled probe showed no signal. Although both miR-195 and -451 demonstrated widespread localization, there appeared to be a non-uniform distribution from the endocardial to epicardial surface, with reduced expression in the outer compact layer of the myocardium (Figure 2). The similar distribution patterns of miR-195 and -451 suggest that both miRs may be concordantly regulated. Under high-powered magnification (200X magnitude) miR-195 and -451 appeared to be concentrated in discrete cytoplasmic foci, the so-called P-bodies where miRNA processing occurs [33]. Notably, there was relatively weak localization of miR-451 within the ventricular chamber, where red blood cells might be expected to collect. Moreover, miR-451 signal intensity was weaker than that of miR-195, suggesting weaker expression.

Finally, we wished to determine the contribution of fibroblast cells to the miR-195 and miR-451 pool in the heart. To do this, preparations of neonatal rat ventricular myocytes (NRVMs) from whole hearts were separated into fibroblast or cardiomyocyte fractions. Staining for sarcomeric actin confirmed the fractionation (Supplemental Figure S2). Next, RNA was isolated from both the NRVM and fibroblast fractions to determine miR-195 and -451 expression. By RT-PCR, NRVMs showed approximately a 2-fold and 28-fold elevation in miR-195 and -451 expression, respectively, over fibroblast expression. The specificity of PCR reactions was verified by gel electrophoresis (Supplemental Figure S2). This data plus the in situ hybridization analyses strongly suggest that miR-195 and -451 are primarily expressed in cardiomyocytes. Again, NRVM expression of miR-195 was approximately 4-fold greater than miR-451 expression.

### Functional Target Suppression of MO25 by miR-195

**Figure 3:**
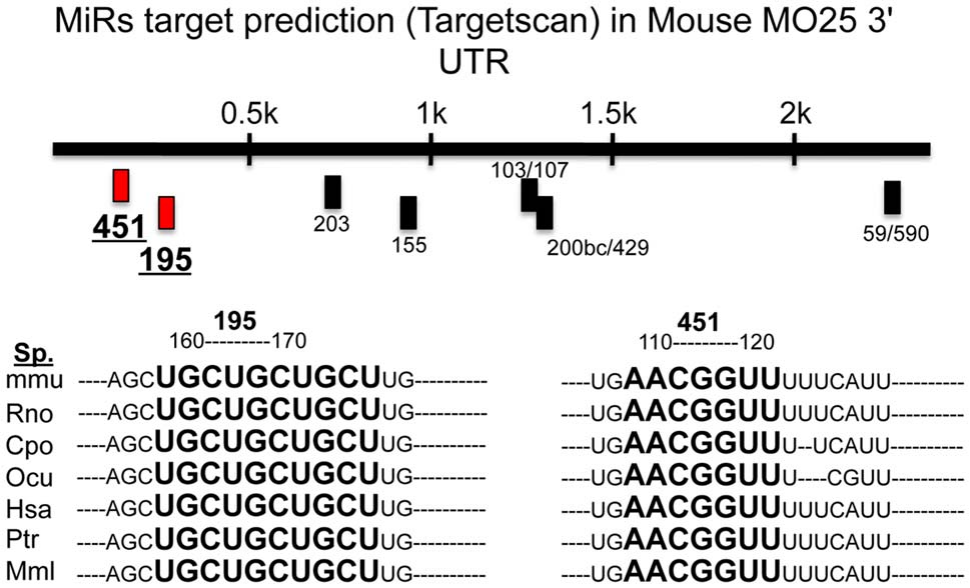
MiRs, including miR-195 and -451, target prediction (Targetscan) in murine MO25 3′ UTR. **Top panel:** Putative binding sites for miR-195 and miR-451 (underlined) are highlighted in red. **Bottom panel:** Sequence alignment (indicated in boldface and large lettering) of putative miR-195 and miR-451 binding sites in 3′ UTR of MO25 of several species, showing a high level of sequence conservation.

Interrogation of the Targetscan 5.1 database revealed a large number of potential miR-195 targets, while only a limited number of conserved targets of miR-451 were predicted. Overall, only two targets were shared by miR-195 and -451: MO25, also known as CAB39, and F-box protein33. MiR-195 and -451 binding sites within MO25 3′UTR are illustrated in Figure 3. The predicted binding site sequences for miR-195 and -451 show high evolutionary conservation within the MO25 3′UTR in different species (Figure 3).

**Figure 4:**
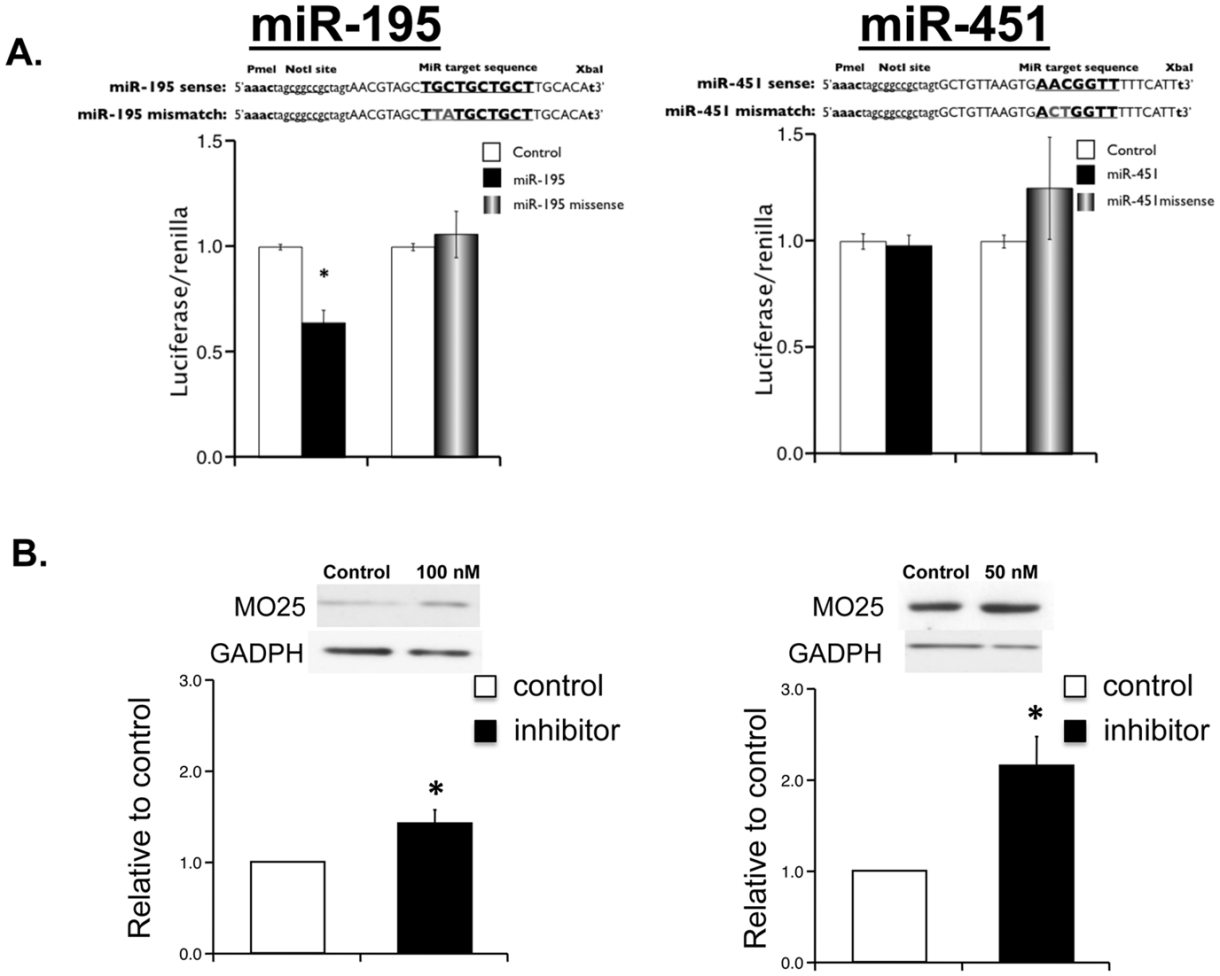
MO25 is a functional target of miR-195 and miR-451. **A:** C2C12 cells transfected with luciferase constructs. MO25 3′ UTR target sequences (as indicated) of miR-195 (**left panel**) or miR-451 (**right panel**). Bar graph representation of luciferase expression in C2C12 cells treated with miR-195 (left panel) or miR-451 (**right panel**) mimics or negative siRNA control. Each bar indicates mean values ± S.E.M. of 3 separate experiments. *P<0.05 compared to WT group. **B:** Increase in expression of MO25 in C2C12 cell by miScript miRNA inhibitor delivered using HiPerfect reagent. AllStars Negative Control siRNA was used as negative control. Each bar indicates mean values ± S.E.M. of 3 separate experiments. *P<0.05 compared to WT group.

The predicated target MO25 was of particular interest due to its role in the LKB1/AMPK signaling pathway. LKB1 complexes with STRAD and MO25 and phosphorylates AMPK, leading to its activation [15]. It was previously demonstrated that, following metabolic stress, glioma cells modulate the LKB1/AMPK pathway by suppressing MO25 expression through an upregulation of miR-451 [27]. Therefore, we hypothesized that MO25 was also a functional target of miR-195. To test whether the predicted sequence within MO25 was a functional target for miR-195, we cloned the miR-195 target sequence within the 3′UTR of MO25 into the pmirGLO Dual-Luciferase Expression Vector. A missense sequence cloned into the same vector served as a negative control. C2C12 cells were transfected with the target or missense sequence vectors and treated with either a miR-195 mimic or negative control siRNA. As shown in Figure 4A, treatment with the miR-195 mimic resulted in significant repression of luciferase activity compared to treatment with the negative control or miR-195 missense siRNA (Figure 4A). Conversely, the miR-451 mimic did not suppress luciferase activity in C2C12 cells transfected with the predicted miR-451 target sequence (Figure 4A). However, treatment of C212 cells with either miR-451 or miR-195 miScript inhibitor significantly increased endogenous C2C12 MO25 expression (Figure 4B), suggesting that MO25 is a functional target of miR-195 and -451. Taken together, these data support MO25 as a functional target of miR-195 and miR-451.

### MO25 Expression and Downstream AMPK Activation in C2C12 Cells

**Figure 5:**
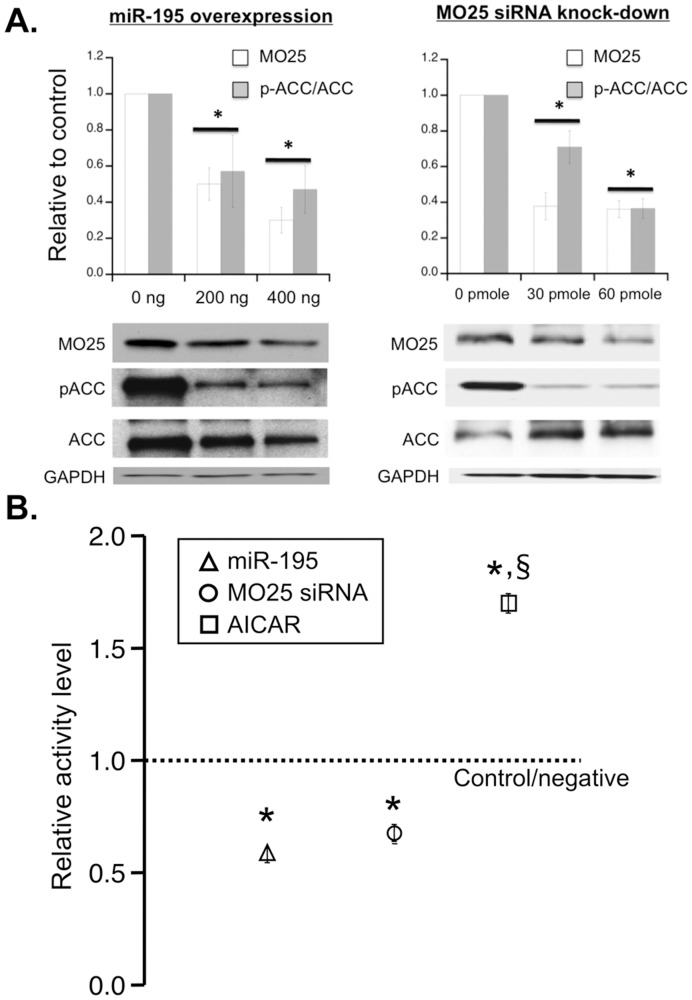
MO25 expression and downstream AMPK signaling in C2C12 cells. **A, Left panel:** Bar graph summary (**top panel**) of Western blots (**bottom panel**) for MO25, acetyl CoA carboxylase (ACC) and phosphorylated ACC (p-ACC) in C2C12 cells transfected with either 0 ng, 200ng or 400ng of miR-195 overexpression constructs. Each bar indicates mean values ± S.E.M. of 3 separate experiments and represents the ratio of total protein to GAPDH or phosphorylated protein to each respective level of total protein following normalization to GAPDH. *P<0.05 compared to 0 ng C2C12 cell group. **A, Right panel:** Bar graph summary (**top panel**) of Western blots (**bottom panel**) for MO25, acetyl CoA carboxylase (ACC) and phosphorylated ACC (p-ACC) in C2C12 cells treated with Stealth RNAi siRNA for miR-195 using HiPerfect reagent. AllStars Negative Control siRNA was used as negative control. Each bar indicates mean values ± S.E.M. of 3 separate experiments and represents the ratio of total protein to GAPDH or phosphorylated protein to each respective level of total protein following normalization to GAPDH. *P<0.05 compared to control group. **B:** miR-195 overexpression and MO25 siRNA knock-down significantly decreased AMPK activities in direct AMPK Kinase assay (Cyclex), While AICAR increased AMPK activities. Each plot indicates mean values ± S.E.M. of 3 separate experiments. *P<0.05 compared to control group; § P<0.05 compared to miR-195 or siRNA knock-down group.

Considering the approximately 8-fold higher levels of miR-195 versus miR-451, we focused on determining whether miR-195 was capable of knocking down MO25 protein expression. Consequently, the effect of miR-195 overexpression on MO25 levels in C2C12 cells was assessed. As demonstrated in Figure 5A, overexpression of miR-195 in C2C12 cells significantly reduced MO25 protein levels. The ratio of phosphorylated Acetyl CoA carboxylase (p-ACC) to total ACC, the downstream target of activated AMPK, was also reduced indicating a reduction in AMPK activity (Figure 5A; left panel). Proficient knock-down of MO25 using siRNA also resulted in reduced AMPK signaling, similar to miR-195 overexpression (Figure 5A; right panel). In addition, both miR-195 overexpression and MO25 siRNA knock-down significantly decreased AMPK activity as measured by phosphorylation of the target peptide in the AMPK activity assay detailed in the methods (Figure 5B). Conversely, treatment of C2C12 cells with 5-aminoimidazole-4-carboxamide riboside (AICAR), which acts as an AMP analog to directly activate AMPK signaling [34], [35], robustly increased AMPK activity (Figure 5B). These data show that expression of miR-195 is sufficient to reduce MO25 proetin levels and downstream AMPK target activation. Moreover, these data also intimate that activation of AMPK activity depends on sufficient MO25 expression. The suggestion is that activation of the AMPK complex depends on proper stoichiometry between LKB1, STRAD, and MO25.

### MO25 Expression and Downstream AMPK Activation in 60-day Male R403Q HCM Hearts

**Figure 6:**
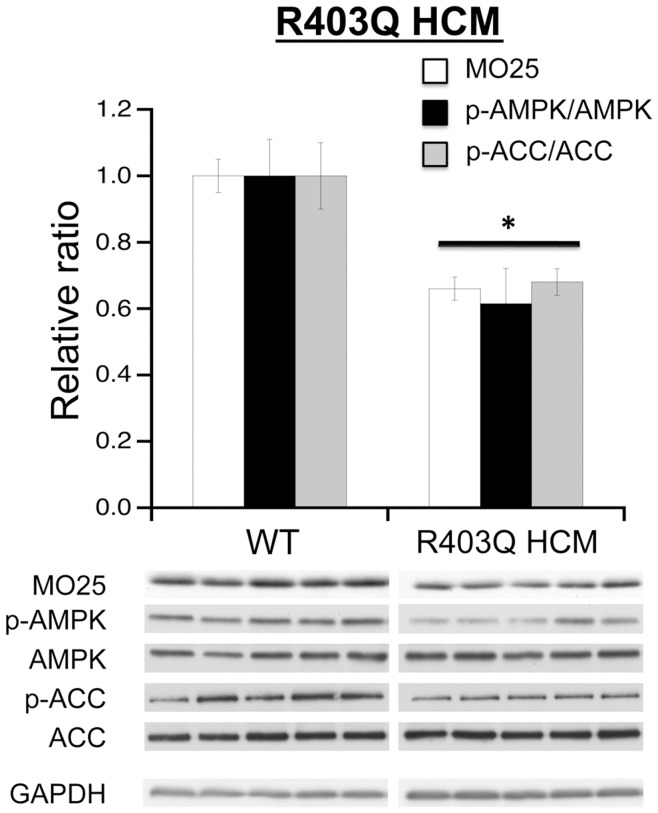
Modification of AMPK signaling axis in R403Q HCM hearts. Bar graph summary (**top panel**) of Western blots (**bottom panel**) for MO25, AMPK, phosphorylated AMPK (p-AMPK), ACC and p-ACC using left ventricular lysates from 60 day male WT or R403Q HCM hearts. Each bar indicates mean values ± S.E.M. (n = 6 hearts) and represents ratio of total protein to GAPDH or phosphorylated protein to each respective level of total protein following normalization to GAPDH. *P<0.05 compared to WT controls.

A previous study by van Rooij et al [6] demonstrates that overexpression of miR-195 is sufficient to induce cardiac hypertrophy and dysfunction in mice. Considering that R403Q HCM male mice develop progressively worsening cardiac disease and demonstrate an early elevation of miR-195 expression, we predicted that elevated miR-195 levels at 60 days would be temporally associated with a decrease in MO25 expression. Moreover, considering the functional role of MO25/STRAD/LKB1 in AMPK signaling, this decrease in MO25 expression should result in a decrease in the AMPK signaling axis. Therefore, we performed Western blot analysis to investigate MO25, p-AMPK, and p-ACC expression levels in 60 day R403Q HCM male hearts. As predicted, the hearts of male R403Q HCM mice demonstrated a significant reduction in MO25 expression compared to WT littermate controls (Figure 6). More importantly, the decrease in MO25 expression was accompanied by a decrease of the p-AMPK(Thr172)/total AMPK ratio as a measure of AMPK activity. The downstream target of activated AMPK (p-AMPK), ACC showed a parallel decrease in phosphorylation (p-ACC). These data indicate that in a mouse model of HCM (R403Q), an elevation of miR-195 expression is accompanied by a decrease in MO25 expression paralleled by a functional repression of the AMPK signaling axis.

**Figure 7:**
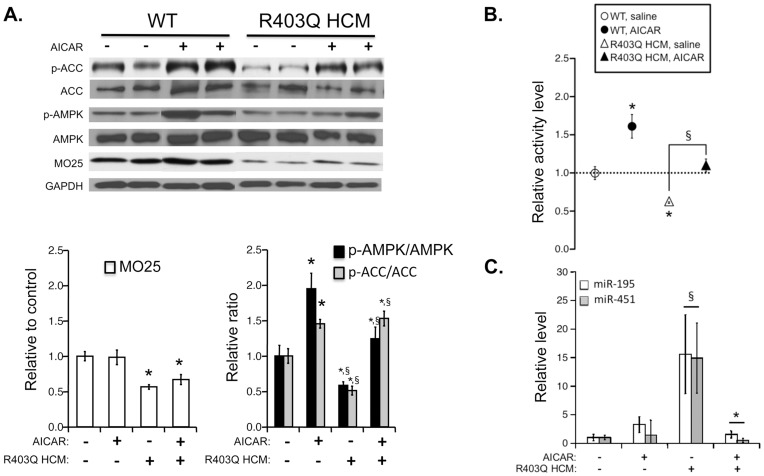
Modification of AMPK signaling axis in R403Q HCM hearts following five-day administration of AICAR. **A:** Bar graph summary (**top panel**) of Western blots (**bottom panel**) for MO25, AMPK, phosphorylated AMPK (p-AMPK), ACC and p-ACC using left ventricular lysates from 60 day male WT or R403Q HCM hearts with or without AICAR administration. Each bar indicates mean values ± S.E.M. (n = 3 hearts) and represents ratio of total protein to GAPDH or phosphorylated protein to each respective level of total protein following normalization to GAPDH. *P<0.05 compared to controls. **B:** AMPK activities in WT or R403Q HCM hearts following five-day administration of AICAR. Each plot indicates mean values ± S.E.M. of 5 separate experiments. *P<0.05 compared to respective control group; § P<0.05 compared to WT control group. **C:** Relative expression of miR-195 and miR-451 in WT or R403Q HCM hearts following AICAR treatment. Expression levels were determined by RT-PCR and illustrated relative to WT saline injected animals. Data are presented as the mean values ± S.E.M. of 4–5 samples. *P<0.05 compared to respective control group; § P<0.05 compared to WT control group.

We wished to further validate that the cellular suppression of AMPK signaling in R403Q HCM hearts was linked to the reduced MO25 expression. We predicted that directly increasing AMPK activity would elevate downstream phosphorylation of ACC even with lowered levels of MO25 protein. Therefore, we treated 60-day R403Q HCM male mice with AICAR to activate AMPK directly, eliminating the requirement for upstream MO25/STRAD/LKB1 target phosphorylation of AMPK [34], [35]. Consistent with 60-day untreated R403Q HCM and WT mice (Figure 6), R403Q HCM mice showed reduced levels of MO25 expression compared to controls whether treated with saline or AICAR (Figure 7A). Again, this resulted in reduced AMPK target activation measured by lower levels of p-AMPK and p-ACC (Figure 7A). Consistent with target activation of AMPK by AICAR, WT AICAR-treated hearts displayed elevated p-AMPK and p-ACC indicative of increased AMPK activity. More importantly, in the context of reduced MO25 expression, 5 days of AICAR treatment in R403Q HCM mice increased AMPK activity as indicated by an elevated p-ACC/ACC ratio over saline-treated R403Q counterparts and WT controls. We also observed a significant increase in AMPK phosphorylation following AICAR treatment, similar to previous studies [36], [37].

To provide functional relevance to the Western blot analysis, we also performed AMPK activity assays using tissue homogenates from the above hearts. Consistent with our Western Blot analysis, AICAR treatment in WT mice resulted in an increase in cardiac AMPK activity (Figure 7B). Again, saline-treated R403Q hearts showed a decrease in AMPK activity similar to the decreased p-AMPK and p-ACC ratios by Western blot. Treatment with AICAR was able to rescue the decrease in AMPK activity observed in R403Q hearts and attenuate AMPK activity to that of WT controls. Taken together, these data imply that the loss of downstream AMPK activity associated with the decrease in MO25 expression is mediated through AMPK. This suggests that the cellular response to increased miR-195 expression coupled with reduced MO25 expression is a suppression of AMPK signaling and that this pathway is functionally relevant in the intact heart.

Finally, to determine any direct effects of AICAR treatment on miR-195 or -451 expression, we also measured miR-195 and miR-451 levels using the above hearts by RT-PCR. As expected, both miR-195 and miR-451 are elevated in R403Q hearts relative to respective WT controls (Figure 7C). We found AICAR treatment in R403Q HCM mice decreased the expression levels of both miR-195 and miR-451. There was no significant change in miR-195 or miR-451 levels in WT mice following AICAR treatment. Interestingly, we also observed a large variation in response to AICAR treatment in HCM mice, which is likely due to our short-term (5-day) injection regimen.

## Discussion

In this study, a candidate screen of micro-RNAs (miRs) in WT versus R403Q HCM hearts revealed a pattern of miR expression that includes the elevation of predicted and novel miR targets. The cardiac disease model used in this study is a model of hypertrophic cardiomyopathy (HCM) that harbors a missense mutation in the α-MyHC gene (R403Q) corresponding to the human mutation in β-MyHC causing HCM [22]. The R403Q model has significantly contributed to our understanding of HCM largely because this particular murine model of human HCM possesses multiple phenotypic similarities with their human counterparts including, (1) histologic and physiological characteristics, (2) course of disease progression, (3) pathological spectrum of disease phenotype, and (4) phenotypic differences between the sexes. More importantly, the R403Q model displays energetic abnormalities such as lowered levels of mitochondrial transcription factor A, nuclear respiratory factor 1, and cytochrome C [25], [26].

The elevated expression of MiR-195 was particularly interesting based on previous findings that overexpression of miR-195 is sufficient to drive a pathological hypertrophic response [6]. In addition, miR-451 is elevated during myocardial infarction and targets the protein of interest, MO25 [27], [38]. Here, the identification of MO25 as a functional target of miR-195 and miR-451 highlights a potential mechanism by which miRs regulate the AMPK signaling axis and potentially induces cardiac hypertrophy and dysfunction. Moreover, these data provide further in vitro support regarding the requirement of proper LKB1/STRAD/MO25 complex formation for full activation of AMPK signaling [39]. In summary, our study illustrates that miR-195 and -451 functionally regulate MO25 expression and that miR-195 is sufficient to suppress MO25 expression and activation of downstream targets of the LKB1/STRAD/MO25 pathway.

It has been established that during the early stages of pathological cardiac hypertrophy, substrate preference in the heart switches to glucose while fatty acid oxidation (FAO) either stays the same or decreases [40]–[42], thereby decreasing the overall energy reserve in the heart [43], [44]. As cardiac disease progresses, the loss of total Cr and PCr results in elevated ADP and AMP and subsequent activation of AMPK [13]. The R403Q HCM model also demonstrates these energetic abnormalities including lower PCr and higher ADP contributing to overall lower levels of ATP, and decreased mitochondrial respiration [26], [45].

Considering the role of the LKB1/STRAD/MO25 complex as an AMPK kinase, we present evidence that miRs, specifically miR-195, can functionally regulate energetic pathways in C2C12 cells. The demonstration that this cellular phenotype was paralleled in cardiac myocytes from a diseased (R403Q HCM) heart suggests a functional role in cardiac disease pathology. In this study, we observe an elevation in miR-195 at 60 and 120 days postnatally that attenuates by 240 days. Although R403Q HCM mice develop hypertrophy by 120 days of postnatal development, the hypertrophy is accompanied by supranormal systolic function but minimal, if any, pathological markers at postnatal 60 days [22], [29]. A strong elevation of miR 208a [6], [46], a known cardiac hypertrophy regulator, is observed at both 60 and 120 days, indicating that hypertrophic progression and cardiac remodeling have started at a very early age. The suggestion from this and previous studies is that the early elevation of miR-195 via a decrease in LKB1/AMPK signaling contributes to the initiation of pathological remodeling by 60 days. It is worth noting that at this same timepoint (60 days), female R403Q HCM mice do not undergo pathological remodeling in the heart and do not show an elevation in miR-195 expression (data not shown).

The finding that overexpression of miR-195 in a transgenic model is sufficient to induce cardiac hypertrophy and dysfunction [6] supports our contention that elevation of miR-195 expression contributes to the R403Q HCM pathology. Similarly, rescue of the cellular phenotype through short-term, pharmacologic activation of AMPK with AICAR illustrates a potential mechanism for the reversal of the HCM disease phenotype. However, the R403Q model of HCM is a progressive disease that requires long-term follow-up in order to assess the efficacy of the AICAR treatment on HCM disease pathology. Yet, it is recently reported that long-term application of Metformin attenuated ventricular hypertrophy induced by pressure overload in a rat model via activation of the AMPK pathway [47]. Overall, the finding that miR-195 expression levels decrease by 240 days illustrates that the progression of HCM pathology requires a complex series of signaling events beyond those regulated by miR-195. Future studies will be aimed at determining whether the cellular rescue of AMPK signaling leads to a reversal of HCM pathology.

This study provides important insight regarding the requirement of MO25 for proper LKB1/STRAD/MO25 complex formation and subsequent activation of AMPK. More importantly, this is the first report showing that functional disruption of this complex can be achieved by miRs in cardiomyocytes. Upstream AMPK activation depends on target phosphorylation by the tumor suppressor LKB1, which must exist in a heterotrimeric complex with STRAD and MO25 in 1∶1∶1 ratio [18], [39], [48]. Previous work in HEK293 cells shows that the absence of either STRAD or MO25 eliminates AMPK activity [39]. In this study, we show that reducing MO25 expression by siRNA knock-down or miR-195 overexpression in C2C12 cells results in a concomitant decrease in relative AMPK activity. The presumption is that suppression of MO25 expression reduces the availability of MO25 available to form the LKB1/STRAD/MO25 heterotrimeric complexes. The net impact is a reduction in relative AMPK activity and downstream target activation. How manipulation of MO25 expression or other components of the heterotrimeric complex impacts complex stoichiometry and/or activity is currently being investigated.

A previous report shows that miR-451 also directly targets MO25 to regulate LKB1/AMPK activity in glioma cells following a metabolic stress [27]. Although the luciferase reporter assay failed to show direct targeting of the putative miR-451 binding site in the MO25 3′ UTR, a miR-451 inhibitor increased MO25 expression in C212 cells suggesting that miR-451 is sufficient to suppress MO25. The significant elevation and presence of miR-451 in R403Q HCM hearts suggests a role in HCM disease progression. Given that miR-195 and miR-451 target MO25, it is likely that these miRs are acting as dual, combinatorial regulators of MO25 and the AMPK pathway.

Our finding that miR-451 is expressed in cardiac tissue represents a significant and potentially novel observation. MiR-451 is selectively expressed in erythroid cells and is regulated by GATA1, a transcription factor essential for hematopoiesis [32]. Although previous reports identified expression of miR-451 in the heart [32], [38], [49], erythroid cell contamination could not be ruled out. We provide direct evidence that miR-451 is expressed in cardiomyocytes (as shown in Figure 2) by in situ hybridization and RT-PCR (Supplemental Figure S2), and in C2C12 cells by RT-PCR (data not shown). Furthermore, a recent study suggests that in cardiomyocytes miR-451 is regulated by GATA4 instead of GATA1 [32].

**Figure 8:**
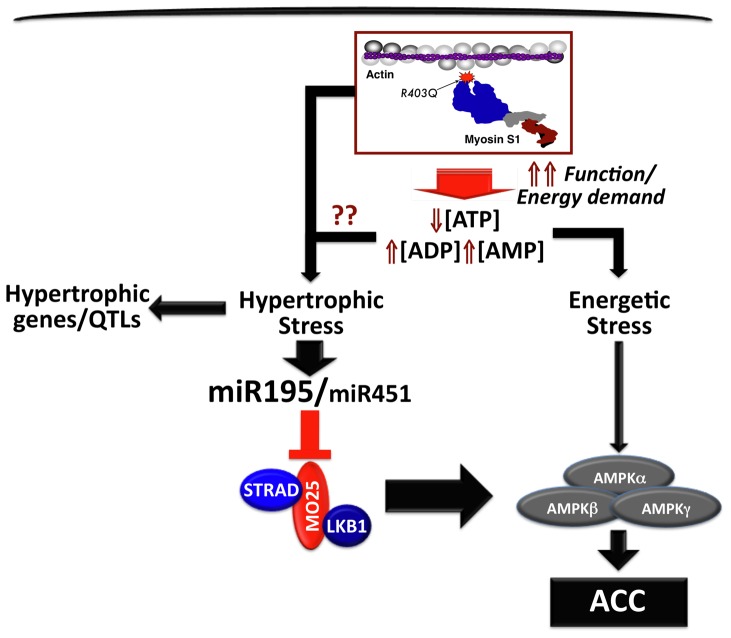
Predicted model of miR-195/mi-R451 regulation of the AMPK signaling axis in R403Q HCM hearts. The functional consequence of the R403Q mutation in the heart is an increase in the relative amount of AMP and ADP. This, in turn, will induce a hypertrophic stress that will elevate expression of miR-195 and miR-451. In addition, the energetic stress caused by this mutation will drive an elevation in AMPK signaling. Based on our findings, the net result is that hypertrophic stress overrides the energetic stress (large arrow) such that miR-195-dependent reduction in MO25 expression leads to a decrease in AMPK signaling indicated by decreased levels of p-ACC.

To conclude, we propose that the R403Q HCM mutation induces a hypertrophic stress leading to the early elevation of miR-195 and miR-451. Through an independent mechanism, R403Q HCM hearts induce an energetic stress that would be predicted to elevate AMPK signaling in order to protect the heart. Indeed, total AMPK protein expression appears to be slightly elevated in R403Q HCM relative to WT hearts at 60 days. However, the net result of the hypertrophic compared to the energetic stress is a suppression of MO25 expression, which in turn leads to a decrease in LKB1 activity and subsequent reduction in AMPK signaling (Figure 8). It is possible that miR-195 and -451 may act as a master energetic “brake” under increases in energetic demand. For example, we have preliminary evidence that shows an increase in cardiac miR-195 expression following 4 weeks of cage wheel exercise. However, this reduction in AMPK signaling exacerbates HCM disease progression. Future studies will be aimed at elucidating the specific role of miR-195 and -451 in the progression of cardiac disease pathology.

## Materials and Methods

### Statement of Ethical Approval

This study was performed in strict accordance with the recommendations in the Guide for the Care and Use of Laboratory Animals of the National Institutes of Health. All of the animals were handled according to approved institutional animal care and use committee (IACUC) protocols (#08–133) of the University of Arizona. The protocol was approved by the Committee on the Ethics of Animal Experiments of the University of Minnesota (Permit Number: 27–2956). All surgery was performed under sodium pentobarbital anesthesia, and every effort was made to minimize suffering.

### Experimental Animals

The experimental murine model has been detailed previously and consists of male mice heterozygous for the mutant α-myosin transgene [22]. All of the animals were handled according to approved IACUC protocols of the University of Arizona. The transgene coding region contained 2 mutations, a point mutation, R403Q, and a deletion of 59 amino acids in the actin-binding site bridged by the addition of 9 nonmyosin amino acids. Hearts of R403Q HCM and WT littermate mice were harvested at 60, 120 and 240 days after birth. Then, the left ventricle was dissected from the whole heart, snap frozen in liquid nitrogen and stored at −80°C for further analysis.

### Real-time PCR

Total RNA was isolated from the left ventricles of WT and R403Q HCM hearts or from neonatal rat cardiomyocytes using the mirVana miRNA isolation kit (Ambion) according to the manufacturer’s protocol. Total and miRNA-specific cDNA was generated using the NCode™ miRNA First-Strand cDNA Synthesis Kit (Invitrogen). Maxima™ SYBR Green qPCR Master Mix (fermentas) was used for real time PCR reactions. U6 RNA was used as an internal control for real time PCR. Sequences of miR and U6 specific primers are listed in Supplemental Table S1.

### Northern Blot Analysis

Northern blots were performed according to a previously described protocol [50]. LNA-modified, 5′ end biotin-conjugated probes for U6 RNA, miR-195 and miR-451 were obtained from Exiqon (Denmark). Bright star BioDect kit (Ambion) was used for the detection of biotinylated probes. U6 snRNA was used as a loading control. All representative Northern blot bands are taken from the same gel but may have been cropped for clarity to illustrate differences in miR expression among the experimental groups.

### *In situ* Hybridization

LNA-modified, 5′ end Digoxin-conjugated probes for scrambled miR control, miR-195 and miR-451 were purchased from Exiqon (Denmark). miRNA in situ hybridization was performed as described at http://geisha.arizona.edu [51].

#### Target predictions and genomic locus search of miR-195 and miR-451

miRBase was used to identify miR-195 and miR-451 loci in the rat genome, and to design real time PCR primers. TargetScan Release 5.1 was used for miR-195 and miR-451 target prediction.

### Luciferase Reporter Assays

For luciferase reporter assays, the pmirGLO Dual-Luciferase Expression Vector (Promega) containing the wild-type or mutated miR target sequence in the MO25 3′UTR was cotransfected with allstars negative control siRNA (Qiagen) or miScript miRNA Mimics (Qiagen) into C2C12 cells. The Dual-Luciferase® Reporter Assay System (Promega) was used for the measurement of luciferase activities. Targeting efficiency was determined by measuring Rinella luciferase levels in a Fluostar Optima plate reader (BMG Labtech) in triplicate.

#### Cell culture and transfection

The C2C12 cell line was obtained from ATCC and cultured in DMEM containing 10% FCS. Luciferase reporter vectors and miR mimics or allstars negative control siRNA (Qiagen) were co transfected into C2C12 cells using the Attractene Transfection Reagent (Invitrogen). The miR-195 expression vector was generously provided by Dr. Eric N. Olsen (UT Southwestern). The miR-195 expression vector was transfected into C2C12 cells using effectene transfection reagent (Invitrogen). Transfection of Stealth RNAi™ siRNA(Invitrogen), or miScript miRNA Inhibitors (Qiagen), or AllStars Negative Control siRNA was accomplished using HiPerfect reagent (Qiagen). 48 hours post-transfection, media were replaced with no glucose DMEM containing no FBS. Cells were harvested at 72 hours post-transfection for further analysis. For the direct measurement of AMPK kinase activities of cultured cells, 10 nM AICAR was added to culture 1 hour before harvest.

### AMPK Kinase Assay

AMPK activity of cultured cells or cardiac tissues was measured using Cyclex AMPK Kinase Assay kit (Cyclex). Briefly, 20 mg of total protein of either cell lysates or supernatant of homogenized tissues diluted in 100 ml kinase buffer was added to and incubated in wells pre-coated with a substrate-peptide corresponding to surrounding mouse IRS-1 S789, which contains the serine residue that can be efficiently phosphorylated by AMPK. An anti-phospho-mouse IRS-1 S 789 monoclonal antibody was used to detect the phosphorylation of the substrate-peptide.

### Neonatal Rat Cardiomyocyte (NRC) and Fibroblast Cell Culture and Immunocytochemistry

Cardiomyocytes were isolated from 1–3 day old neonatal rats and maintained as described [52]. Briefly, hearts of neonatal rats were minced and digested in Earle’s salt solution, without Ca2+ and Mg2+, containing 0.125% pancreatin. After discarding the initial two 15-min digests, cells were collected and resuspended in Ham’s F-12K medium containing 250 pg/ml fetuin, 10 mg/ml bovine serum albumin, 20 pg/ml ascorbic acid, 100 units/ml penicillin, and 100 pg/ml streptomycin (pH 7.4). The cells were preplated on 100 mm petri dish for 3 h to allow fibroblast attachment. The preplate, which contains mostly fibroblast (>95%), was used for fibroblast analysis. Unattached cells, which are mostly cardiomyocytes, were pelleted and resuspended in the culture medium for cardiomyocyte analysis. Forty-eight hours after plating, cardiomyocytes or fibroblasts were either used for RNA extraction or immunocytochemistry. A standard immunocytocemistry procedure was performed using anti-sarcomere α-actinin monoclonal antibody (Sigma) at a dilution of 1∶500 for staining of cardiomyocytes.

### Administration of AICAR

5-amino-4-imidazole-1-β-carboxamide ribofuranoside (AICAR) was purchased from Toronto Chemicals. AICAR was administered i.p. (0.5 mg/g body weight in sterile saline at a volume of 500 µl/mouse) daily for 5 days. Mice in the control group received 500 µl of PBS. Upon completion of the 5-day injection protocol, hearts were harvested 2 hours after the last injection.

### Western Blot Analysis

Heart or cell lysates were prepared by mechanical disruption in a protein extraction buffer (in mmol/L): Tris(hydroxymethyl)-aminomethane (50); ethylene glycol-bis(b-aminoethyl ether)-N,N,N’,N’-tetraacetic acid (EGTA) (0.5); EDTA (1); dithiothreitol (DTT) (0.5) (pH 7.0). The buffer also contained (in mmol/L) leupeptin (0.1), pepstatin (0.1), phenylmethylsulfonyl fluoride (0.1) to prevent non-specific proteolysis and sodium pyrophosphate (1) and sodium vanadate (1) to prevent non-specific phosphorylation or dephosphorylation, respectively. SDS-PAGE was performed followed by transfer to a membrane (polyvinylidene difluoride [PVDF]) for Western Blot analysis. All antibodies were obtained from Cell Signaling Technology, except anti-GAPDH which was obtained from Abcam. In addition, prior to immunoblotting, all membranes were stained with Ponceau S acid red and quantified for total protein. Next, total protein measured by Ponceau S was compared to GAPDH expression for equal loading. All representative Western Blot bands are taken from the same gel but may have been cropped for clarity to illustrate differences among the experimental groups. Data reported as averages ± SEM.

### Data and Statistical Analysis

Results are presented as mean ± SEM. An ANOVA followed by a Tukey’s post-hoc test or a student’s t test was performed to compare differences between mean values. P values of <0.05 were considered statistically significant.

## Supporting Information

Figure S1 **Candidate miRNA screen in R403Q HCM and WT hearts using RT PCR.** Bar graph representation of the fold change in miR expression in male R403Q HCM and WT hearts at 60, 120 and 240 days. All values are compared to the miR expression level of WT contols after normalization to U6 expression. Each bar is the average of 3 independent RT-PCR experiments from unique animals.
(TIF)

Figure S2 **Cardiomyocyte-specific expression of miR-195 and miR-451.** Neonatal ventricular cardiomyocytes (NRVMs) were pre-plated in order to generate a separate pool of NRVMs and fibroblasts. From either the NRVM or fibroblast pool, RT-PCR was performed using sequence-specific primers for miR-195 or miR-451. **A.** **Top panel:** immunohistochemistry was performed using an anti-sarcomere actinin monoclonal antibody to distinguish between NRVMs (**left; positive staining**) and fibroblasts (**right; negative staining**). The fibroblast pool showed little to no positive staining for sarcomeric actinin. **Bottom panel:** Agarose gel electrophoresis of RT-PCR. Visualization of RT-PCR products from the RT-PCR reactions. **B.** Bar graph representation of the fold change a based on RT-PCR results in miR-195 or -451 expression from NRVMs and fibroblast pools. MiR expression levels in fibroblast pool was compared to miR expression from NRVM pool.
(TIF)

Table S1 **Reverse primer sequences used in detection of miRNAs by real time PCR.** The reverse complement sequences of mature mouse miRNA sequences were used as primers. The universal primer of NCode miRNA kit (Invitrogen) was used as forward primer for all the real time PCR reactions.
(DOCX)

## References

[1] Esquela-Kerscher A, Slack FJ (2006). Oncomirs - microRNAs with a role in cancer.. Nat Rev Cancer.

[2] Glazov EA, Pheasant M, Nahkuri S, Mattick JS (2006). Evidence for control of splicing by alternative RNA secondary structures in Dipteran homothorax pre-mRNA.. RNA Biol.

[3] Hammond SM (2006). RNAi, microRNAs, and human disease.. Cancer Chemother Pharmacol.

[4] Ikeda S, Kong SW, Lu J, Bisping E, Zhang H (2007). Altered microRNA expression in human heart disease.. Physiol Genomics.

[5] Matkovich SJ, Van Booven DJ, Youker KA, Torre-Amione G, Diwan A (2009). Reciprocal regulation of myocardial microRNAs and messenger RNA in human cardiomyopathy and reversal of the microRNA signature by biomechanical support.. Circulation.

[6] van Rooij E, Sutherland LB, Liu N, Williams AH, McAnally J (2006). A signature pattern of stress-responsive microRNAs that can evoke cardiac hypertrophy and heart failure.. Proc Natl Acad Sci U S A.

[7] van Rooij E, Marshall WS, Olson EN (2008). Toward microRNA-based therapeutics for heart disease: the sense in antisense.. Circ Res.

[8] Port JD, Sucharov C (2010). Role of MicroRNAs in cardiovascular disease: therapeutic challenges and potentials.. J Cardiovasc Pharmacol.

[9] Small EM, Olson EN (2011). Pervasive roles of microRNAs in cardiovascular biology.. Nature.

[10] van Rooij E, Sutherland LB, Qi X, Richardson JA, Hill J (2007). Control of stress-dependent cardiac growth and gene expression by a microRNA.. Science.

[11] Montgomery RL, Hullinger TG, Semus HM, Dickinson BA, Seto AG, et al. Therapeutic inhibition of miR-208a improves cardiac function and survival during heart failure.. Circulation.

[12] Dyck JR, Hopkins TA, Bonnet S, Michelakis ED, Young ME (2006). Absence of malonyl coenzyme A decarboxylase in mice increases cardiac glucose oxidation and protects the heart from ischemic injury.. Circulation.

[13] Tian R, Musi N, D’Agostino J, Hirshman MF, Goodyear LJ (2001). Increased adenosine monophosphate-activated protein kinase activity in rat hearts with pressure-overload hypertrophy.. Circulation.

[14] Wong AK, Howie J, Petrie JR, Lang CC (2009). AMP-activated protein kinase pathway: a potential therapeutic target in cardiometabolic disease.. Clin Sci (Lond).

[15] Hawley SA, Boudeau J, Reid JL, Mustard KJ, Udd L (2003). Complexes between the LKB1 tumor suppressor, STRAD alpha/beta and MO25 alpha/beta are upstream kinases in the AMP-activated protein kinase cascade.. J Biol.

[16] Woods SC, Gotoh K, Clegg DJ (2003). Gender differences in the control of energy homeostasis.. Exp Biol Med (Maywood).

[17] Anderson RA (1998). Chromium, glucose intolerance and diabetes.. J Am Coll Nutr.

[18] Baas AF, Boudeau J, Sapkota GP, Smit L, Medema R (2003). Activation of the tumour suppressor kinase LKB1 by the STE20-like pseudokinase STRAD.. Embo J.

[19] Shaw RJ, Kosmatka M, Bardeesy N, Hurley RL, Witters LA (2004). The tumor suppressor LKB1 kinase directly activates AMP-activated kinase and regulates apoptosis in response to energy stress.. Proc Natl Acad Sci U S A.

[20] Hardie DG, Hawley SA, Scott JW (2006). AMP-activated protein kinase–development of the energy sensor concept.. J Physiol.

[21] Winder WW, Holmes BF, Rubink DS, Jensen EB, Chen M (2000). Activation of AMP-activated protein kinase increases mitochondrial enzymes in skeletal muscle.. J Appl Physiol.

[22] Vikstrom KL, Factor SM, Leinwand LA (1996). Mice expressing mutant myosin heavy chains are a model for familial hypertrophic cardiomyopathy. PG - 556–67.. Mol Med 2.

[23] Olsson MC, Palmer BM, Leinwand LA, Moore RL (2001). Gender and aging in a transgenic mouse model of hypertrophic cardiomyopathy.. Am J Physiol Heart Circ Physiol.

[24] Stauffer BL, Konhilas JP, Luczak ED, Leinwand LA (2006). Soy diet worsens heart disease in mice.. J Clin Invest.

[25] Watson PA, Reusch JE, McCune SA, Leinwand LA, Luckey SW (2007). Restoration of CREB function is linked to completion and stabilization of adaptive cardiac hypertrophy in response to exercise.. Am J Physiol Heart Circ Physiol.

[26] Lucas DT, Aryal P, Szweda LI, Koch WJ, Leinwand LA (2003). Alterations in mitochondrial function in a mouse model of hypertrophic cardiomyopathy.. Am J Physiol Heart Circ Physiol.

[27] Godlewski J, Nowicki MO, Bronisz A, Nuovo G, Palatini J (2010). MicroRNA-451 regulates LKB1/AMPK signaling and allows adaptation to metabolic stress in glioma cells.. Mol Cell.

[28] Esau C, Davis S, Murray SF, Yu XX, Pandey SK (2006). miR-122 regulation of lipid metabolism revealed by in vivo antisense targeting.. Cell Metab.

[29] Olsson MC, Palmer BM, Stauffer BL, Leinwand LA, Moore RL (2004). Morphological and functional alterations in ventricular myocytes from male transgenic mice with hypertrophic cardiomyopathy.. Circ Res.

[30] Vikstrom KL, Bohlmeyer T, Factor SM, Leinwand LA (1998). Hypertrophy, pathology, and molecular markers of cardiac pathogenesis.. Circ Res.

[31] Zhang X, Wang X, Zhu H, Zhu C, Wang Y (2010). Synergistic effects of the GATA-4-mediated miR-144/451 cluster in protection against simulated ischemia/reperfusion-induced cardiomyocyte death.. J Mol Cell Cardiol.

[32] Dore LC, Amigo JD, Dos Santos CO, Zhang Z, Gai X (2008). A GATA-1-regulated microRNA locus essential for erythropoiesis.. Proc Natl Acad Sci U S A.

[33] Liu J, Valencia-Sanchez MA, Hannon GJ, Parker R (2005). MicroRNA-dependent localization of targeted mRNAs to mammalian P-bodies.. Nat Cell Biol.

[34] Corton JM, Gillespie JG, Hawley SA, Hardie DG (1995). 5-aminoimidazole-4-carboxamide ribonucleoside. A specific method for activating AMP-activated protein kinase in intact cells?. Eur J Biochem.

[35] Hardie DG, Carling D, Carlson M (1998). The AMP-activated/SNF1 protein kinase subfamily: metabolic sensors of the eukaryotic cell?. Annu Rev Biochem.

[36] Williamson DL, Bolster DR, Kimball SR, Jefferson LS (2006). Time course changes in signaling pathways and protein synthesis in C2C12 myotubes following AMPK activation by AICAR.. Am J Physiol Endocrinol Metab.

[37] Sun Y, Connors KE, Yang DQ (2007). AICAR induces phosphorylation of AMPK in an ATM-dependent, LKB1-independent manner.. Mol Cell Biochem.

[38] Bostjancic E, Zidar N, Glavac D (2009). MicroRNA microarray expression profiling in human myocardial infarction.. Dis Markers.

[39] Zeqiraj E, Filippi BM, Deak M, Alessi DR, van Aalten DM (2009). Structure of the LKB1-STRAD-MO25 complex reveals an allosteric mechanism of kinase activation.. Science.

[40] Nascimben L, Ingwall JS, Lorell BH, Pinz I, Schultz V (2004). Mechanisms for increased glycolysis in the hypertrophied rat heart.. Hypertension.

[41] Degens H, de Brouwer KF, Gilde AJ, Lindhout M, Willemsen PH (2006). Cardiac fatty acid metabolism is preserved in the compensated hypertrophic rat heart.. Basic Res Cardiol.

[42] Barger PM, Kelly DP (2000). PPAR signaling in the control of cardiac energy metabolism.. Trends Cardiovasc Med.

[43] Kalsi KK, Smolenski RT, Pritchard RD, Khaghani A, Seymour AM (1999). Energetics and function of the failing human heart with dilated or hypertrophic cardiomyopathy.. Eur J Clin Invest.

[44] Osorio JC, Stanley WC, Linke A, Castellari M, Diep QN (2002). Impaired myocardial fatty acid oxidation and reduced protein expression of retinoid X receptor-alpha in pacing-induced heart failure.. Circulation.

[45] Spindler M, Saupe KW, Christe ME, Sweeney HL, Seidman CE (1998). Diastolic dysfunction and altered energetics in the alphaMHC403/+ mouse model of familial hypertrophic cardiomyopathy.. J Clin Invest.

[46] Callis TE, Pandya K, Seok HY, Tang RH, Tatsuguchi M (2009). MicroRNA-208a is a regulator of cardiac hypertrophy and conduction in mice.. J Clin Invest.

[47] Zhang CX, Pan SN, Meng RS, Peng CQ, Xiong ZJ (2010). Metformin attenuates ventricular hypertrophy by activating the AMP-activated protein kinase-endothelial nitric oxide synthase pathway in rats.. Clin Exp Pharmacol Physiol.

[48] Boudeau J, Baas AF, Deak M, Morrice NA, Kieloch A (2003). MO25alpha/beta interact with STRADalpha/beta enhancing their ability to bind, activate and localize LKB1 in the cytoplasm.. Embo J.

[49] Caruso P, MacLean MR, Khanin R, McClure J, Soon E (2010). Dynamic changes in lung microRNA profiles during the development of pulmonary hypertension due to chronic hypoxia and monocrotaline.. Arterioscler Thromb Vasc Biol.

[50] Pall GS, Hamilton AJ (2008). Improved northern blot method for enhanced detection of small RNA.. Nat Protoc.

[51] Bell GW, Yatskievych TA, Antin PB (2004). GEISHA, a whole-mount in situ hybridization gene expression screen in chicken embryos.. Dev Dyn.

[52] Gustafson TA, Bahl JJ, Markham BE, Roeske WR, Morkin E (1987). Hormonal regulation of myosin heavy chain and alpha-actin gene expression in cultured fetal rat heart myocytes.. J Biol Chem.

