# Supplementary figures and images for: Micro-RNA-195 and -451 Regulate the LKB1/AMPK Signaling Axis by Targeting MO25
Source: PLoS One. 2012 Jul 23;7(7):e41574. doi: 10.1371/journal.pone.0041574 (PMC3402395; doi:10.1371/journal.pone.0041574)

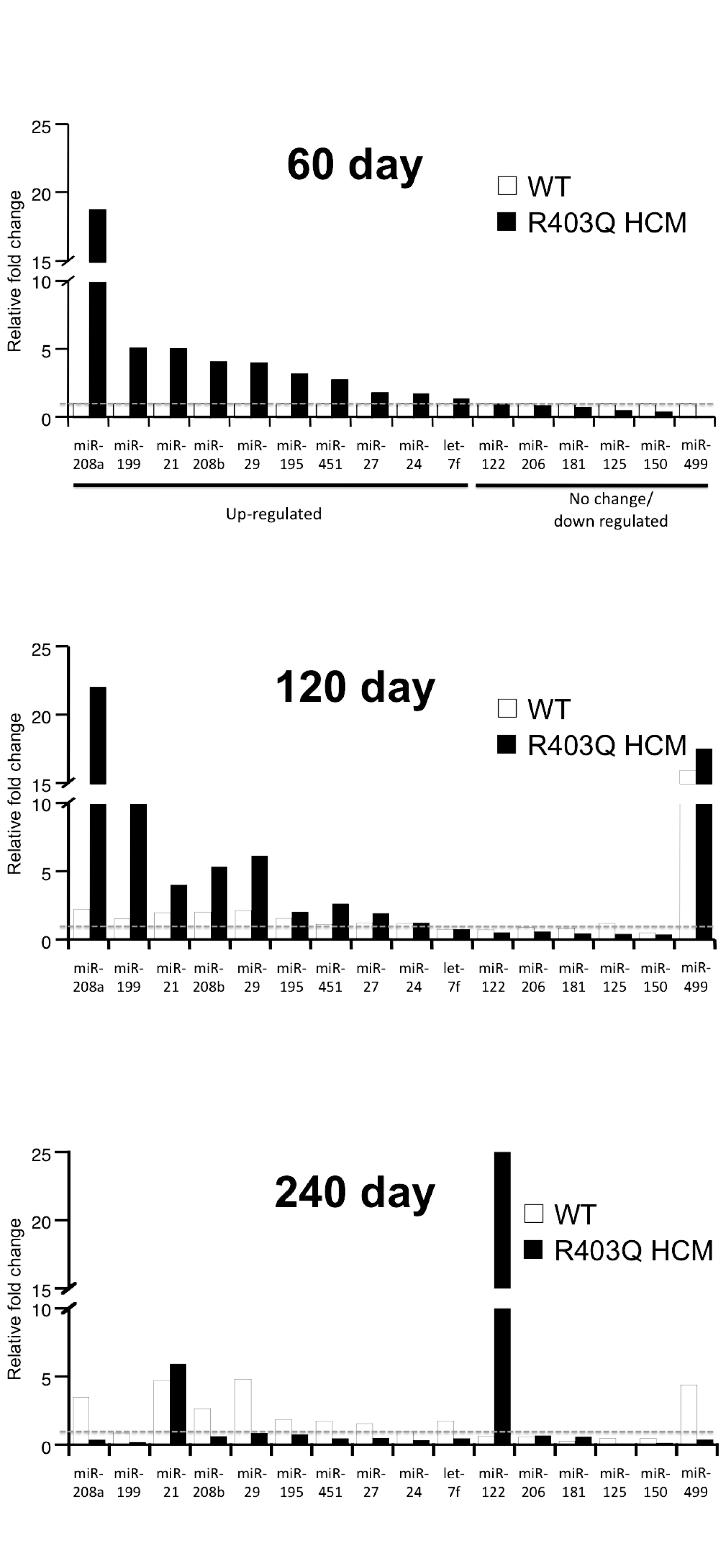

Supplement: Figure S1 — Candidate miRNA screen in R403Q HCM and WT hearts using RT PCR. Bar graph representation of the fold change in miR expression in male R403Q HCM and WT hearts at 60, 120 and 240 days. All values are compared to the miR expression level of WT contols after normalization to U6 expression. Each bar is the average of 3 independent RT-PCR experiments from unique animals. (TIF) [file pone.0041574.s001.tif]

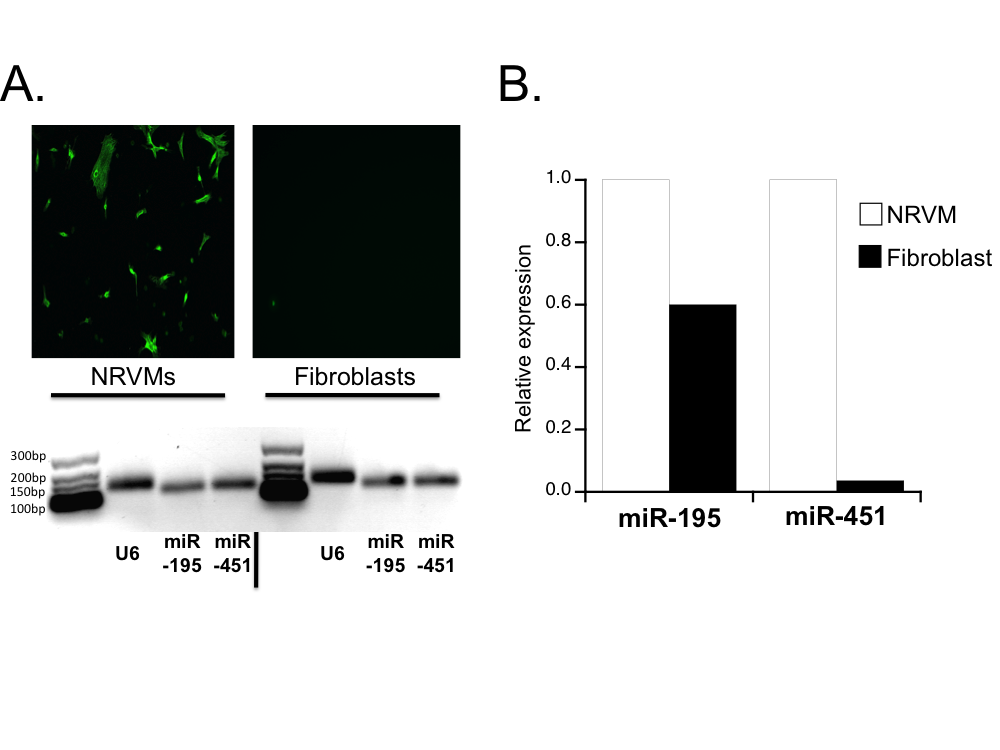

Supplement: Figure S2 — Cardiomyocyte-specific expression of miR-195 and miR-451. Neonatal ventricular cardiomyocytes (NRVMs) were pre-plated in order to generate a separate pool of NRVMs and fibroblasts. From either the NRVM or fibroblast pool, RT-PCR was performed using sequence-specific primers for miR-195 or miR-451. A. Top panel: immunohistochemistry was performed using an anti-sarcomere actinin monoclonal antibody to distinguish between NRVMs (left; positive staining) and fibroblasts (right; negative staining). The fibroblast pool showed little to no positive staining for sarcomeric actinin. Bottom panel: Agarose gel electrophoresis of RT-PCR. Visualization of RT-PCR products from the RT-PCR reactions. B. Bar graph representation of the fold change a based on RT-PCR results in miR-195 or -451 expression from NRVMs and fibroblast pools. MiR expression levels in fibroblast pool was compared to miR expression from NRVM pool. (TIF) [file pone.0041574.s002.tif]
