# Supplementary material for: Micro-RNA-195 and -451 Regulate the LKB1/AMPK Signaling Axis by Targeting MO25
Source: PLoS One. 2012 Jul 23;7(7):e41574. doi: 10.1371/journal.pone.0041574 (PMC3402395; doi:10.1371/journal.pone.0041574)
Supplement: Table S1 — Reverse primer sequences used in detection of miRNAs by real time PCR. The reverse complement sequences of mature mouse miRNA sequences were used as primers. The universal primer of NCode miRNA kit (Invitrogen) was used as forward primer for all the real time PCR reactions. (DOCX) [file pone.0041574.s003.docx]

**Supplemental Table S1**. Sequences of RT-PCR primers for the miRNA candidate screen

| **MiRNA** | **Nucleotide Sequence** |
| --- | --- |
| miR-21 | Uagcuuaucagacugauguuga |
| miR-23a | aucacauugccagggauuucc |
| miR-23b | atcacattgccagggattacc |
| miR-24 | Uggcucaguucagcaggaacag |
| miR-27a | Uucacaguggcuaaguuccgc |
| miR-125b | Ucccugagacccuaacuuguga |
| miR-195 | Uagcagcacagaaauauuggc |
| miR- 199a | cccaguguucagacuaccuguuc |
| miR-214 | Acagcaggcacagacaggcagu |
| miR-217 | tactgcatcaggaactgattgga |
| miR-29c | Uagcaccauuugaaaucgguua |
| miR-93 | caaagtgctgttcgtgcaggtag |
| miR 133a | Uuugguccccuucaaccagcug |
| miR-150 | Ucucccaacccuuguaccagug |
| miR-181b | aacattcattgctgtcggtgggt |
| miR-1 | tggaatgtaaagaagtatgtat |
| miR-133 | tttggtccccttcaaccagctg |
| miR-206 | tggaatgtaaggaagtgtgtgg |
| u6 | CACGAATTTGCGTGTCATCCTT |
| miR-122 | Tggagtgtgacaatggtgtttg |
| let-7c | tgaggtagtaggttgtatggtt |
| miR-451 | aaaccgttaccattactgagtt |
| miR-208a | ataagacgagcaaaaagcttgt |
| miR-208b | ataagacgaacaaaaggtttgt |
| miR-499 | ttaagacttgcagtgatgttt |
| miR-486 | tcctgtactgagctgccccgag |
